# Carotenoids from Halophilic Archaea: A Novel Approach to Improve Egg Quality and Cecal Microbiota in Laying Hens

**DOI:** 10.3390/ani14233470

**Published:** 2024-12-01

**Authors:** Xufeng Dou, Guodong Zhang, Hao Tang, Xiaoxue Chen, Beibei Chen, Yuxia Mei, Haihong Jiao, Min Ren

**Affiliations:** 1 Key Laboratory of Conservation and Utilization of Biological Resources in the Tarim Basin, Alar 843300, China; douxufeng69@163.com (X.D.);; 2 College of Life Science and Technology, Tarim University, Alar 843300, China; 3 College of Animal Science and Technology, Tarim University, Alar 843300, China; 18009484694@163.com (G.Z.);; 4 College of Life Science and Technology, Huazhong Agricultural University, Wuhan 430000, China

**Keywords:** carotenoids, egg quality, *Halalkalicoccus paucihalophilus*, antioxidant capacity, cecal microbiota

## Abstract

Carotenoids from different sources have different structures and functions, and their dietary components benefit the health of various organisms. The effects of halophilic Archaea-derived C50 carotenoids on poultry egg quality and gut microbiota remain largely unexplored. In this study, we isolated a carotenoid-secreting strain of *Halalkalicoccus paucihalophilus*, TRM89021, from the Pamir Plateau. We characterized the carotenoid pigments produced by this strain; the major components were bacterioruberin and its derivatives. The effects of these carotenoids on the egg quality and cecal microbiota composition of hens were investigated. Compared to the basal diet group (BDG), supplementation with carotenoids in the carotenoids-supplemented diet group (CDG) resulted in significantly lower a* and b* scores at week 5 and lower b* scores and Haugh units at week 2, while egg strength and weight were higher. CDG also showed increased yolk antioxidant capacity, higher glutathione peroxidase levels, and significantly lower catalase levels (*p* < 0.05). Plasma analysis revealed elevated total bilirubin and aspartate aminotransferase levels, along with reduced inorganic phosphorus levels in the CDG (*p* < 0.05). No significant differences in cecal microbiota diversity were observed between the groups at any taxonomic level. This result suggests that halophilic archaea-derived carotenoids have the potential to be used as natural feed supplements to improve egg quality. Our study provides a theoretical basis for applying archaea-derived carotenoids in poultry diets.

## Simple Summary

Carotenoids are important dietary component. Different sources of carotenoids differ in structure and function, and they all have potential benefits. However, the effects of C50 carotenoids produced by halophilic archaea on poultry egg quality and gut microbiota remain unexplored. In this study, the carotenoid-producing Archaea species *Halalkalicoccus paucihalophilus* TRM89021 was isolated from the Pamir Plateau. Feeding Hotan Black hens with diets containing additional carotenoids from *H. paucihalophilus* improved eggshell redness and yolk antioxidant levels. Although no significant change was observed in the diversity of the cecal microbiota, *Bacteroidota* were more dominant in the treatment group. An increase in *Bacteroidota* in the chicken’s gut can lead to more effective digestion of food, better absorption of nutrients, and maintenance of gut health. This suggests that carotenoids from halophilic archaea can be used as natural feed additives to improve egg quality and modulate gut microbiota in poultry. This study provides a novel method for adding carotenoids to poultry diets and a theoretical basis for their application.

## 1. Introduction

Carotenoids, a significant group of natural pigments, are known for their role in bestowing vibrant hues to plants, algae, and various microorganisms. Moreover, they possess crucial biological functions, including antioxidant activity [1,2,3]. These pigments are isoprenoid polymers with 40–50 carbon atoms. To date, over 700 distinct natural carotenoids have been discovered. Among microorganisms, halophilic archaea constitute a unique class of carotenoid-producing organisms, including the red strain *Haloferax volcanii* [4] and the newly isolated *Salinarchaeum acidiphilum*, obtained from the solar salt fields of Sfax, Tunisia [5], both of which are capable of producing carotenoids. These microorganisms typically thrive in marine and inland salt environments, including salt lakes and salt mines [6,7,8,9]. They are also commonly found in the soils of the Pamir Plateau [10,11]. These strains are uniquely capable of synthesizing C50 carotenoids [12]. Carotenoid C50 exhibits superior antioxidant activity compared with C40 carotenoids, including β-carotene and astaxanthin [13,14]. Additionally, C50 surpasses non-carotenoid antioxidants like tocopherols, butylated hydroxytoluene, and ascorbic acid in its antioxidant capabilities [15]. Employing high-performance liquid chromatography (HPLC) and HPLC–mass spectrometry (HPLC-MS) techniques, researchers have determined that halophilic archaea produce both C40 and C50 carotenoids [16,17]. The research indicates that after fermenting red halophilic archaea, the subsequent isolation and purification of pigments can be obtained using column chromatography and thin layer chromatography. Characterization through visible light spectroscopy, Raman spectroscopy, nuclear magnetic resonance (NMR), and mass spectrometry has revealed that cell membranes are enriched with carotenoids, such as bacterioruberin, monoanhydrobacterioruberin, and bisanhydrobacterioruberin [18,19]. Furthermore, carotenoids such as β-carotene and lycopene, which are present in lower concentrations, can serve as precursors for the synthesis of additional carotenoids by halophilic archaea [20].

Carotenoids are renowned for their roles in the development of plants, algae, and various microorganisms. In addition to their coloration, they possess major biological functions, including antioxidant activities. Supplementing poultry diets with carotenoids has been demonstrated to enhance both egg yield and quality, in addition to having a positive effect on the gut microbiota of birds [21,22]. For instance, the addition of microorganism-derived astaxanthin to the feed of laying hens influenced the antioxidant capacity of plasma and improved the color of egg yolks [23,24]. In addition, it can enhance egg yolk coloring [24,25]. Intake of plant-derived carotenoids significantly modulates animal intestinal and fecal microbiota [26,27,28]. The impact of microbial-derived pigments on gut microorganisms in chickens remains undocumented.

A noticeable gap exists in the research regarding the efficacy of *Halophilic archaea* carotenoids in poultry feed, particularly in Hotan Black chickens. The Hotan Black chicken, a breed of considerable economic importance, is characterized by its black plumage, robust disease resistance, capacity to flourish on rough feed, and adaptability to hot environments [29]. Primarily utilised for meat and egg production, the Hotan Black chicken was recognized in the geographical indication agreement for agricultural products in Xinjiang in 2013 [30], indicating its regional importance. This breed responds differently to feed additives compared with other species [31,32]. Consequently, investigating the use of *Halophilic archaea* carotenoids in the diet of Hotan Black chickens could yield new strategies for enhancing egg quality and intestinal health; however, this area requires further scientific research and empirical evidence.

In this study, we isolated and obtained pigmented halophilic archaea from Pamir Plateau soil. Carotenoids derived from this microorganism were added to the feed of Hotan Black chickens, and their effects on egg yield, egg quality, and cecum microbiota composition were evaluated. This pioneering study introduced a novel carotenoid additive, derived from halophilic archaea, into laying hens’ feed, contributing to a broader range of options for feed additives.

## 2. Materials and Methods

### 2.1. Halophilic Archaea Isolation and Identification

Soil samples collected from the Pamir Plateau were stored at −4 °C [11]. Soil sample classification, archaeal isolation, and identification were performed based on the methodology described by Bu et al. [11]. Briefly, Pamir Plateau soil samples (1 g) were diluted in 9 mL of 15% sterile saline solution, used to inoculate NOM media, and incubated at 37 °C for 30 d. The NOM medium composition (per liter) was as follows: yeast extract 0.05 g, fish peptone 0.25 g, sodium pyruvate 1.0 g, KCl 5.4 g, K_2_HPO_4_ 0.36 g, CaCl_2_ 0.25 g, NH_4_Cl 0.25 g, MgSO_4_·7H_2_O 0.25 g, MgCl_2_·6H_2_O 23.0 g, NaCl 150.0 g, and agar 20 g, and pH was adjusted to 7.0. Individual colonies were selected and purified repeatedly (three times). Genomic DNA was extracted and subjected to 16S rRNA gene amplification (Table S1), using a reaction mixture comprising EasyTaq 0.2 μL, EasyTaq Buffer 2 μL, dNTPs 1.5 μL, forward primers 1 μL, reverse primers 1 μL, DNA 50 ng, and ddH_2_O to a final volume of 20 μL. The primers used were 20F (5′-ATTCCGGTTGATCCTGCC-3′) and 1452R (5′-AGGAGGTGATCCAGCCGC-3′). The PCR products exhibiting positive bands, as identified by 1% (*w*/*v*) agarose gel electrophoresis, were sequenced bidirectionally by Beijing Tsingke Biotech Co., Ltd. (Beijing, China) and Sangon Biotech (Shanghai) Co., Ltd. (Shanghai, China) Sequences were assembled using SeqMan software (Version: 7.1.0) and analyzed for similarity against the Ezbiocloud database (https://www.ezbiocloud.net/) (accessed on 25 September 2024) to ascertain their taxonomic affiliation. Pigment-producing halophilic archaea were frozen and preserved in glycerol tubes at −80 °C (NaCl concentration of 15% (*w*/*v*)) for subsequent use in pigment extraction.

### 2.2. Carotenoids Analysis

Carotenoids were extracted from the red halophilic archaea using conventional methods [33]. After culturing the red halophilic archaea, a 500 mL aliquot of the culture was centrifuged at 12,000 rpm for 5 min at room temperature. The supernatant was decanted to obtain a pellet. Subsequently, 40 mL pure water was added to the pellet. The cell lysate was mixed with a solvent mixture in which the ratio of cell lysate to trichloromethane to methanol was 1:1:2 (*v*/*v*/*v*), and the mixture was transferred to a clean conical flask. The flask was shaken at 150 rpm for 4.5 h at room temperature to facilitate extraction. Pure water was added to the mixture until phase separation occurred, and the mixture was allowed to stand at room temperature for 4 h for complete delamination. The red organic layer was collected using a separatory funnel and concentrated using a rotary evaporator with the water bath temperature set at 30 °C and the rotor speed maintained at 60 rpm (Haydorf Instruments Co., Ltd., Shanghai, China). The crude pigment extract was then dissolved in methanol and transferred to a 10 mL centrifuge tube, and the supernatant was stored at −20 °C for further analysis. The ultraviolet–visible spectral analysis was conducted using a Thermo Scientific Evolution 201 UV-Vis Spectrophotometer (Thermo Fisher Scientific—Shanghai, China). Ultraviolet absorption spectra were recorded in the range of 350 to 600 nm [33]. Furthermore, carotenoids from high-carotenoid-producing strains were analyzed using ultra-performance liquid chromatography (UPLC) [34,35] (ACQUITY UPLC/VION IMS QTOF MS, Waters Corporation, Milford, MA, USA).

Absorbance was measured at 495 nm using a methanol blank. Carotenoid yield was calculated using the following formula [35,36]:

(1) $$Carotenoid\;yield\;(mg/L)=\frac{A\times D\times V1}{0.16\times V2}$$

where C represents the total carotenoid yield (mg/L); A is the absorbance at the maximum wavelength (λ = 495 nm); D is the dilution factor; V1 is the total volume of the extract (unit: L); 0.16 represents the average specific absorption coefficient for carotenoids at 495 nm (unit: L/(mg·cm) or L/(μmol·cm)); and V2 is the volume of the culture medium (unit: L).

High carotenoid-producing strains were inoculated into a sterile fermenter for cultivation over a period of 5 days (silent oil-free air compressor, Shanghai Top Stability Machinery Co., Ltd., Shanghai, China; 100 L fermenter, Shanghai Baoxing Biochemical Equipment Co., Shanghai, China) After the cultivation period, the carotenoids were extracted following the aforementioned method. Finally, the carotenoid powder in the rotavapor flask was scraped out; note that methanol was no longer used for dissolution. The end result was the acquisition of carotenoid powder.

### 2.3. Animal Experimental Design and Management

The experimental protocol adhered to the animal welfare guidelines of the College of Life Sciences and Technology, Tarim University. It also complied with the code of practice for the safe use of feed additives (Ministry of Agriculture Bulletin No. 2625) [37]. We randomly divided 50 late-laying-stage Hotan Black chickens (300 d old) into two groups: a carotenoid-supplemented diet group (CDG) and a basal diet group (BDG), each with five replicates, with five chickens in each replicate. The BDG was fed a basal diet, whereas the CDG was fed a basal diet supplemented with 100 mg/kg of carotenoids extracted from halophilic archaea. (By conducting a pre-test, we learned that 100 mg/kg of carotenoids was more favorable for egg quality.) The carotenoids mentioned were in the form of the obtained carotenoid powder referred to in Section 2.2. Throughout the experiment, the chickens had ad libitum access to water. The basal diet was fed three times a day at regular intervals (morning, midday, and evening) with 500 g per replicated group of chickens per feeding.

We recorded daily feed remaining and daily egg weights at weeks 2 and 5 to calculate the daily feed consumption and egg mass. Then, we calculated the average feed consumption and egg mass for this period. The feed conversion ratio was calculated as the ratio of total feed consumption to produced egg mass during the period [38]. To ensure optimal living conditions, chickens were housed in layered cages with permanent ventilation, exposure to natural light, and regular coop cleaning. The energy content of the basal diet was formulated according to the nutritional requirements specified by the National Research Council for laying hens (1994) [39]. The feeding experiment spanned 5 weeks. The basal feed was procured from Xinjiang Tiankang Feed Co., Ltd. (Urumqi, China), and its detailed nutritional composition is presented in Table 1.

### 2.4. Egg Quality

Egg production was compared between week 2 and week 5. At weeks 2 and 5, three freshly laid eggs were randomly selected from each replicate every day for further analysis. The egg shape dimensions, specifically length and width, were measured using a digital caliper IP54 (Shanghai Deystar Tools Co., Ltd., Shanghai, China). The weight of each egg was determined using an electronic balance (ML204, Mettler Toledo Instruments (Shanghai) Ltd., Shanghai, China). The color of the eggshells was evaluated using an SC-10 eggshell color tester (Beijing Tianxiang Feiyu Technology Co., Ltd., Beijing, China), which quantified the L*, a*, and b* values to assess brightness, redness, and yellowness, respectively. Eggshell strength was measured using a KQ-1A eggshell strength tester (Beijing Tianxiang Feiyu Technology Co., Ltd., Beijing, China). The height of the albumen was determined using an Egg White Height Determination EQ-1A Mini device (Beijing Tianxiang Feiyu Technology Co., Ltd., Beijing, China). The color of the egg yolk was determined using an Egg Yolk Colorimetric Fan, ranging from 1 (yellow) to 15 (red). We calculated the Haugh unit value to assess the quality of the eggs. The formula for the Haugh unit is $HU=100\log\;[H-\frac{\sqrt{G}\;(30W^{0.37}-100)}{100}+1.9]$, where HU stands for Haugh unit, G is the gravitational constant (32.2), H is the observed height of the albumen (in mm), and W is the observed weight of the egg (in g)) [40].

During the experiment, three fresh eggs were randomly selected at 3 d intervals from each replicate, and the antioxidant capacity of egg yolks was determined using total antioxidant capacity (T-AOC), superoxide dismutase (SOD), malondialdehyde (MDA), glutathione peroxidase (GSH-Px), and catalase (CAT) kits (Suzhou Grace Biotechnology Co., Ltd., Suzhou, China).

### 2.5. Plasma Biochemical and Cecal Microbiota

The chickens were fasted for 24 h before the experiment, and water was available ad libitum. Fresh blood was collected from under the wing using a disposable venous blood sampling needle and venous blood sampler (containing sodium heparin), and the blood was stored at 4 °C. Nine chickens were randomly selected from each of the experimental and the control groups, and a scalpel was used to sever the jugular vein. The chickens were dissected, and the cecal contents were swiftly placed into cryovials, which were immediately submerged in liquid nitrogen.

The blood of the 9 treated laying hens was divided into three subgroups, each consisting of blood from 3 chickens. Blood samples from each subgroup were mixed to form a single blood sample. Blood samples from the control group were treated similarly, and three pooled blood samples were obtained. Fresh blood was centrifuged (4 °C, 3000 rpm, 10 min) (high-speed cryo-centrifuge, Thermo Fisher Scientific China Co., Ltd., Shanghai, China) and plasma was collected and analyzed using a fully automated multifunctional biochemical analyzer (SMT-120VP Chengdu Smarter Science and Technology Co., Ltd., Sichuan, China). The biochemical traits included albumin (ALB), inorganic phosphorus (PHOS), total protein (TP), glucose (GLU), amylase (AMY), aspartate aminotransferase (AST), total bilirubin (TB), urea nitrogen (BUN), creatine kinase (CK), albumin–globulin ratio* (A/G*), and globulin* (GLOB*) content.

The samples were transported on dry ice to Shanghai Meiji Biomedical Technology Co., and subjected to full-length bacterial community diversity sequencing (16S rRNA amplicon sequencing).

### 2.6. Statistical Analysis of Data

The egg quality data obtained in this study were subjected to an independent samples *t*-test using SPSS 27.0, and the data results were expressed as mean ± standard (x ± SE). *p* < 0.05 indicated a significant difference. Phylogenetic trees were constructed using MEGA-X software (Version: 10.2.2). Carotenoid production was expressed in mg/L, egg weight and feed remaining in g, eggshell strength in kg/m^2^, and eggshell thickness in mm. According to the antioxidant capacity assay kit, the unit of CAT was µmol/min/g, the unit of GSH-Px was nmol/min/g, the unit of MDA was nmol/g, the unit of SOD was U/g, T-AOC units were µmol Trolox/g, GLOB*, ALB, TP were in g/L, AMY, AST, CK in U/L, and GLU, BUN, and PHOS were in mmol/L. Amplicon sequencing was in OTU.

## 3. Results

### 3.1. Isolation, Purification, and Identification of Pigment-Producing Halophilic Archaea

**Figure 1:**
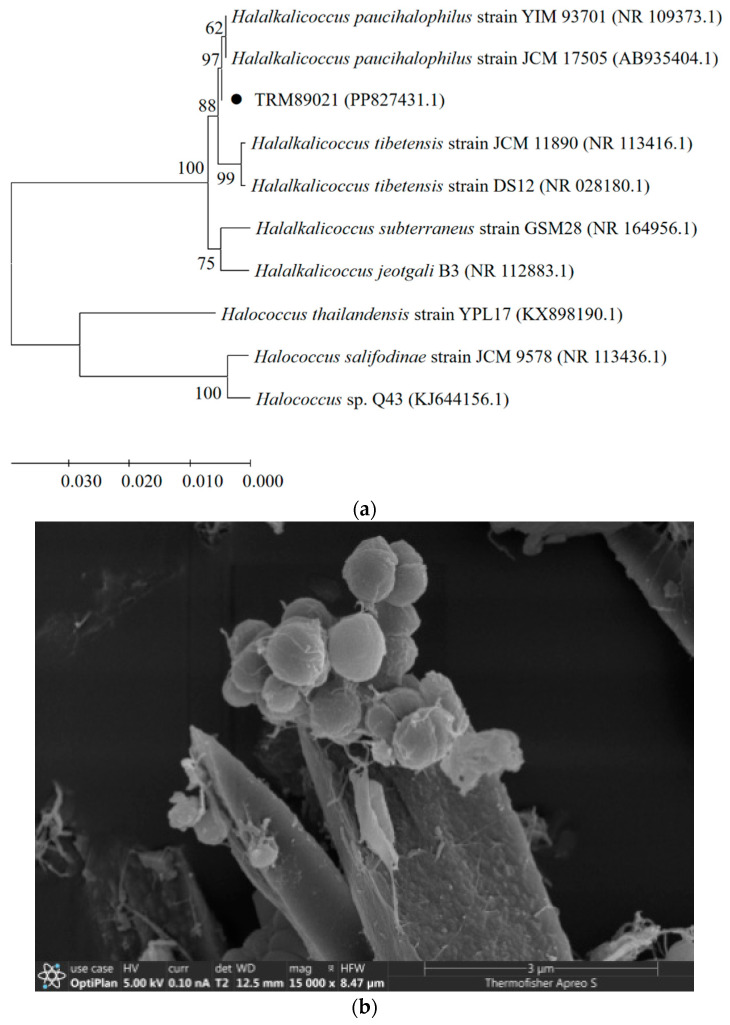
(**a**) Phylogenetic tree of TRM89021. Neighbor-joining tree constructed based on 16S rRNA gene sequences showing the phylogenetic relationships between strain TRM89021 and related taxa. Bootstrap values over 50% are shown on the nodes as percentages of 1000 replicates. *Halococcus thailandensis* YPL17 (KX898190), *Halococcus salifodinae* JCM9578 (NR113436), and *Halococcus* sp. Q43 (KJ644156) were used as an outgroup. (**b**) Scanning electron microscope image. The diameter of the sphere is about 1 µm.

*H. paucihalophilus* TRM89021 (accession number: PP827431) was obtained from soil samples collected from the Pamir Plateau. Colonies of the strain appeared red and exhibited an opaque, smooth, round, and raised morphology. TRM89021 is Gram-negative (G^−^). Scanning electron microscopy revealed spherical cells. An NJ tree based on the 16S rRNA gene (1404 bp) showed that strain TRM 89021 clustered tightly with *H. paucihalophilus* JCM17505 and YIM93701, with strong bootstrap support (Figure 1).

### 3.2. Carotenoid Principal Component Analysis

**Figure 2:**
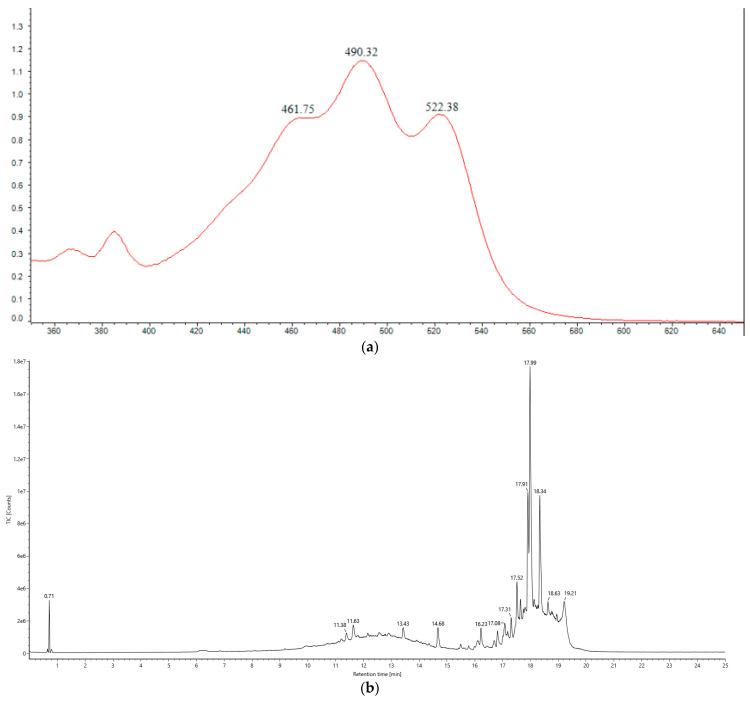

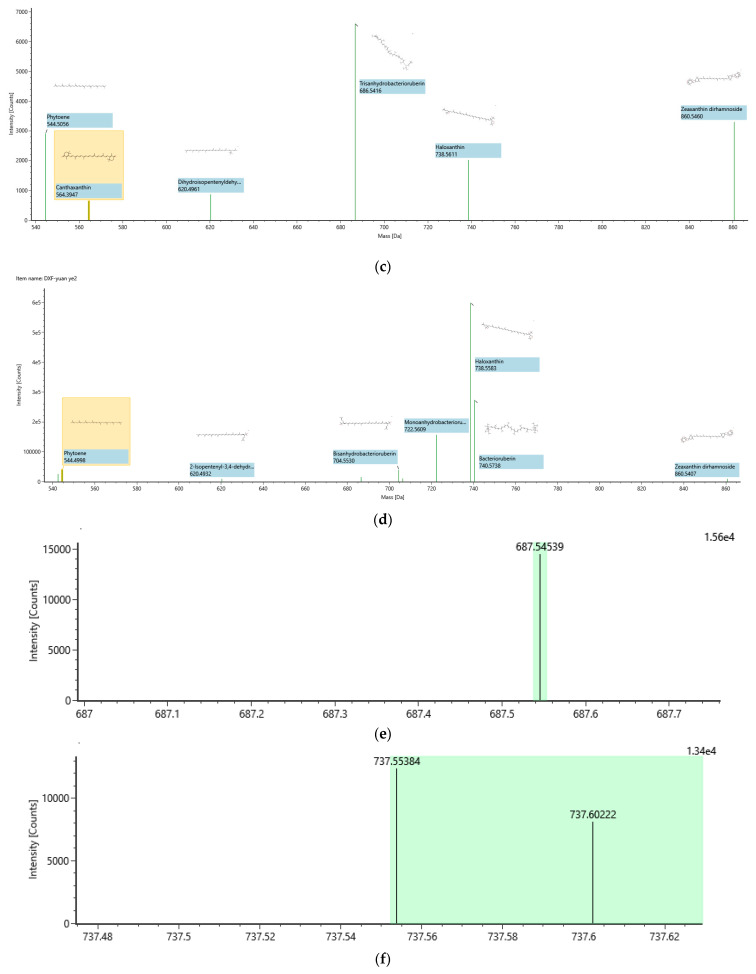

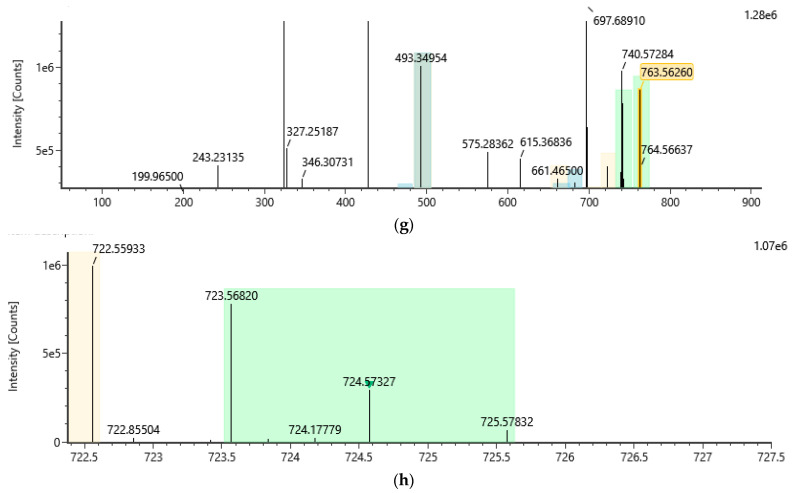
(**a**) UV scanning spectra of TRM89021 carotenoids. The top right corner of Figure 3 shows the results for the bacterial broth obtained from the fermentation of this strain. The figure shows the scanned spectrum of the carotenoid extract at an absorbance of 350–600, and three peaks can be observed at 461 nm, 490 nm and 522 nm, in line with the characteristic “three fingers”; (**b**) ACQUITY UPLC analysis of carotenoid extract of *H. paucihalophilus* TRM89021, scan mass: 50–2000 m/z, scan time 0.2 s, collision energy: 6–45 eV, column temperature: 30 °C; (**c**) detection mode: POSITIVE (positive ion mode); (**d**) detection mode: NEGATIVE (negative ion mode); (**e**) trisanhydrobacterioruberin; (**f**) haloxanthin; (**g**) bacterioruberin; (**h**) monoanhydrobacterioruberin. Figures (**e**–**h**) are all C50 carotenoids.

The UV-Vis absorption spectrum of the crude methanolic extract of the pigment showed distinct peaks at 461 nm, 490 nm, and 522 nm, indicative of the characteristic “three-finger” signature of C50 carotenoids [41]. This observation led to the preliminary identification of these pigments as carotenoids. The UPLC chromatogram of the carotenoid extract from *H. paucihalophilus* TRM89021, along with the ACQUITY UPLC/VION IMS QTOF MS response plot, confirmed that the major constituents of the extract were bacterioruberin and its derivatives, as demonstrated by the chromatographic profiles (Figure 2). The fermentation yield of carotenoids from the strain TRM89021 was 20 mg/L.

### 3.3. Effects of Dietary Carotenoids Extracts on Egg Quality in Hotan Black Chickens

Significant increases in the a* score, egg strength, and egg weight were observed at week 2 compared to week 5 in the BDG group (*p* < 0.05). In the CDG group, the L* score increased significantly and the a* score decreased from week 2 to week 5 (*p* < 0.05). Comparing the two groups, the CDG had significantly lower a* and b* scores at week 5. At week 2, the b* score, Haugh units, and feed conversion ratio were significantly lower, and egg strength and weight were significantly higher than those of the BDG (*p* < 0.05). No significant differences were observed in the other measured indices: eggshell thickness, egg-shaped index, and yolk color (*p* > 0.05) (Table 2).

Comparing the eggs from the CDG at week 2 with those at week 5, a significant decrease was observed in CAT (*p* < 0.01) and there were significant increases in GSH-Px and T-AOC content in the yolks at week 2 *(p* < 0.001). Eggs in the BDG group at week 5 were significantly higher than T-AOC at week 2. Comparing the two groups with each other, the CDG group had significantly lower GSH-Px at week 2 and significantly higher T-AOC capacity (*p* < 0.05) (Table 3).

### 3.4. Impact of Pigment Extracts on Plasma Biochemical Parameters in Laying Hens

Our findings, presented in Table 4, indicate that supplementation with carotenoids led to significant increases in plasma total bilirubin and aspartate aminotransferase levels (*p* < 0.05) and a significant decrease in inorganic phosphorus levels (*p* < 0.05) in the CDG compared with the BDG.

### 3.5. Effects of Dietary Carotenoids on Cecal Microbiota in the Hotan Black Chickens

**Figure 3:**
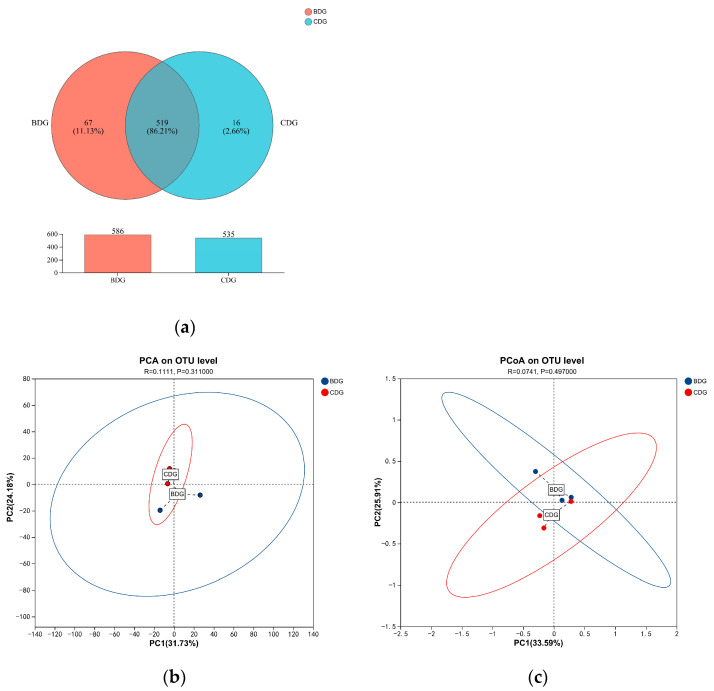
(**a**) Comparison of operational taxonomic units (OTUs) among groups; (**b**) PCA plot based on Bray–Curtis distance, illustrating the separation of bacterial communities between the CDG and BDG groups. Each dot represents a sample, with red indicating the BDG group and blue the CDG group. The percentage of variance explained by each principal component is indicated on the axes. The results show a *p* value of 0.3110, indicating no significant difference; (**c**) PCoA plot derived from the same distance matrix, visualizing the similarity and dissimilarity in microbial community composition. Ellipses represent the 95% confidence intervals for each group. Statistical significance was determined by ANOSIM, with a *p* value of 0.4970, also indicating no significant difference. *p* > 0.05 indicates that the difference between groups is not significant.

Cluster analysis was conducted on clean reads of all samples and sequences were clustered into operational taxonomic units (OTUs). In total, 602 OTUs were identified, each representing a specific microbial species; 535 and 586 OTUs were present in the dietary carotenoid and the control groups, respectively (Figure 3). The species annotation results revealed 155 species, of which 141 were present in the CDG and 153 in the BDG. Carotenoid supplementation did not significantly impact the alpha diversity indices (ACE, Sobs, Simpson, Shannon, coverage, and Chao) of the cecal microbiota OTUs in laying hens (*p* = 0.4469, *p* = 0.3172, *p* = 0.6769, *p* = 0.2271, *p* = 0.1806, and *p* = 0.4131, respectively, Table 5). β-diversity analysis, including PCA based on Bray–Curtis dissimilarity and PCoA, revealed no significant differences in the overall species composition of cecal microbiota between the groups (*p* > 0.05).

**Figure 4:**
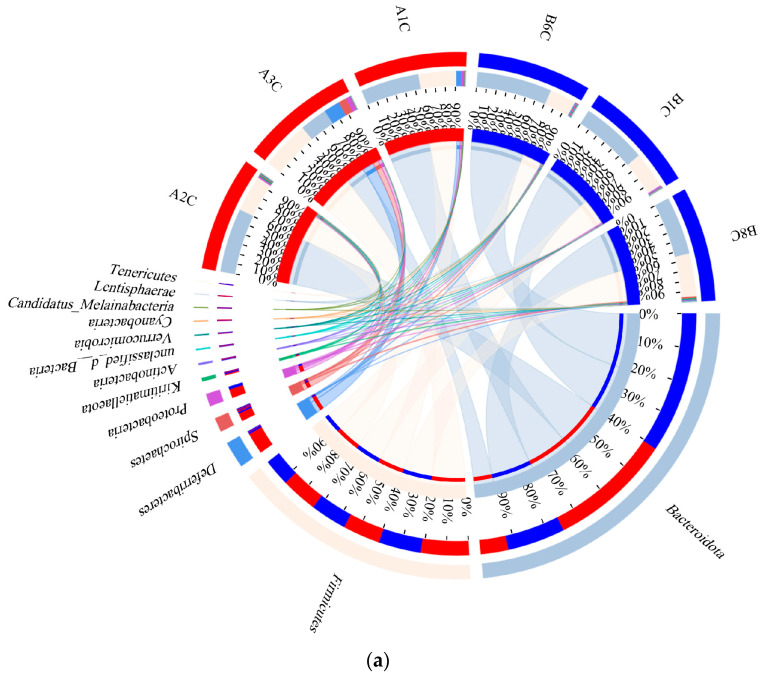

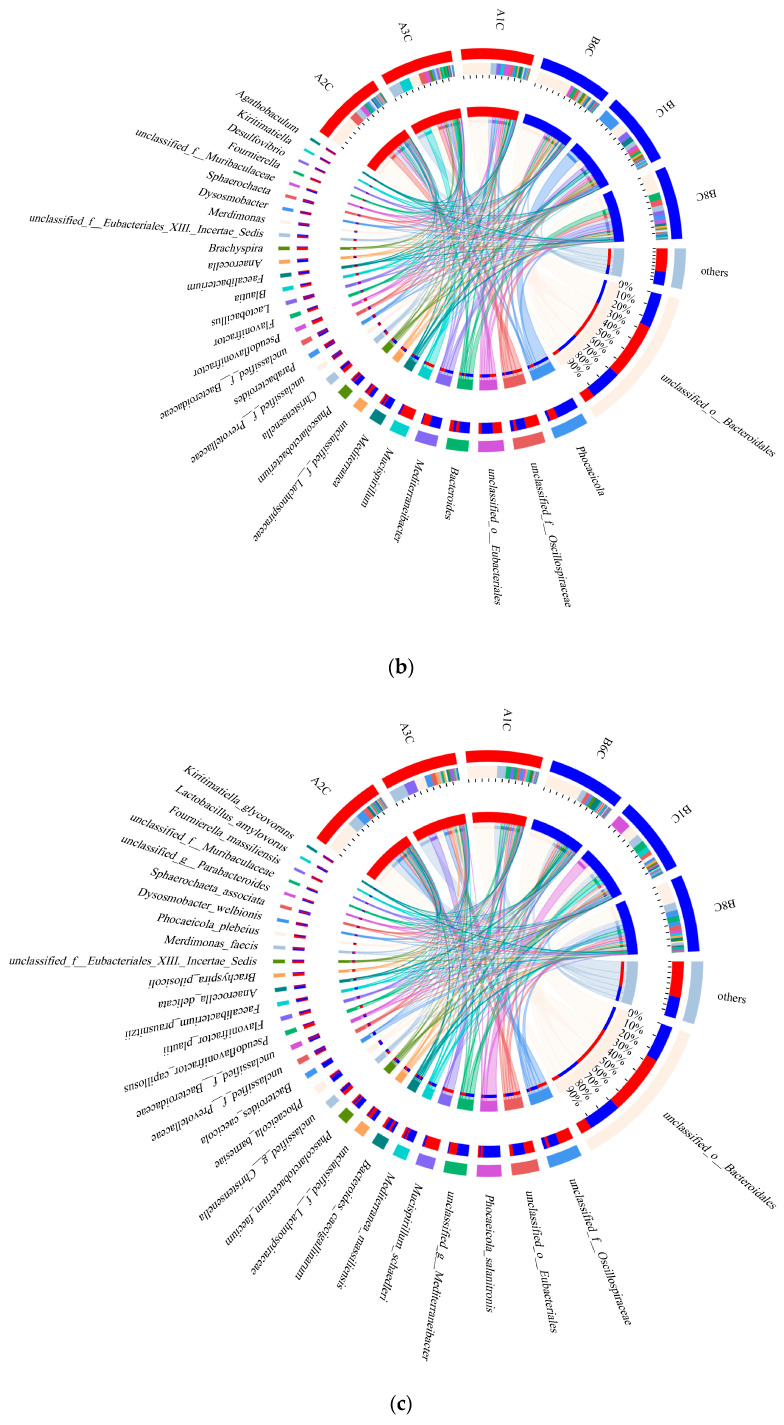

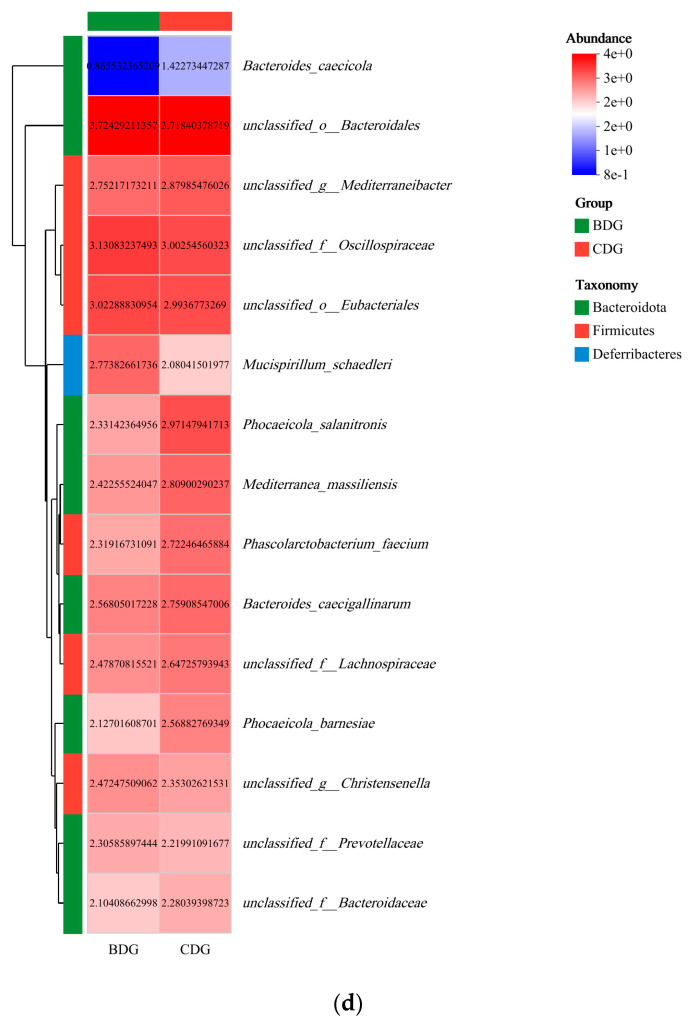
Comparative analysis of microbial community composition between the BD and CD groups: (**a**) phylum level, highlighting the variations in species composition; (**b**) genus level, detailing the differences in species composition; (**c**) species level, examining the nuances in species composition; (**d**) an intuitive visualization of the compositional and abundance profiles of the BDG and CDG bacterial communities, along with their distribution at the species level. Samples A1C, A2C, and A3C represent the CDG group, whereas B1C, B6C, and B8C represent the BDG group.

Regarding species composition, *Bacteroidota* was the predominant phylum in the cecal flora, representing 62.90% and 47.33% of the total CDG and BDG samples, respectively. The second most abundant phylum was *Firmicutes*, comprising 33.40% and 38.65% of the community in the CDG and BDG, respectively. The dominant genera and species were unclassified *Bacteroidales* and non-culturable bacteria, representing 33.03% and 32.60%, respectively (Figure 4 and Figure S1). Additionally, *Phocaeicola coprophilus* and *Paraprevotella clara* occurred only in the CDG, as did 14 species, including *Bacteroides uniformis*, *Ligilactobacillus aviaries*, *Latilactobacillus sakei* (Figure S2). At the phylum, order, family, genus, and species levels, no significant differences in microbial composition were observed between the CDG and BDG (*p* > 0.05). At the genus level, *Phocaeicola* abundance was higher in the CDG (12.04%) than in the BDG (3.82%); the species-level analysis showed that *Phocaeicola salanitronis* in the CDG (8.84%) was more abundant than in the BDG (1.61%) (*p* > 0.05) (Figure S3) (Reference Species Classification Database: nt_v20221012/16s_bacteria).

## 4. Discussion

### 4.1. Isolation, Purification and Identification of Pigment-Producing Halophilic Archaea

Typically, halophilic archaea require a salt concentration of at least 15% to achieve peak growth [42]. Halophilic microorganisms are potential pigment producers [43], with bacteriocins being the predominant carotenoids in halophilic archaea [44]. Pigment production by halophilic archaea is a defence mechanism against environmental stresses, since produced pigments protect halophilic archaea from various external stresses, including high salinity and intense ultraviolet radiation [45]. The literature suggests that halophilic archaea can be isolated from several high-salt environments using a dilution plating technique [46,47,48]; this includes red halophilic archaea [49,50]. Soil samples from the Pamir Plateau contained numerous halophilic archaea, as determined by 16S rRNA amplicon sequencing [10]. In this study, *H. paucihalophilus* TRM89021 was isolated from soil samples collected from the Pamir Plateau using the dilution–coating-plate method. The isolation of halophilic archaea from the Pamir Plateau provides new insights into the study of halophilic microorganisms. These microorganisms are not only prevalent in the high-salt environments previously reported but are also notably abundant in the high-altitude regions of the Pamir Plateau. This discovery reveals the rich resources of halophilic archaea in the Pamir Plateau and suggests that they may play a notable role in the ecosystem balance and biogeochemical cycles of the region.

### 4.2. Carotenoid Principal Component Analysis

Liquid chromatography–mass spectrometry (LC-MS), specifically the HPLC-APCI-MS/MS method, is commonly utilized for the identification of carotenoids [51,52]. For instance, *Halorubrum* sp. strain BS2 was observed to produce carotenoids identified as bacterioruberin and bisanhydrobacterioruberin by HPLC and LC-MS analyses [52]. Analysis of the carotenoids produced by *Halorubrum tebenquichense* SU10 using ultra-performance liquid chromatography electrospray ionisation–tandem mass spectrometry (UPLC-ESI-MS/MS) revealed the presence of bacterioruberin along with several of its derivatives, such as mono-, di-, and tri-hydroxybacterioruberin, and various cis-isomers of bacterioruberin [35]. Using UV, HPLC, and TLC methods, the primary carotenoid components in seven species of halophilic archaea, including *Halogeometricum rufum*, *Halogeometricum limi*, and *Haladaptatus litoreus* were identified as C50 carotenoids: bacterioruberin and its derivatives (mono- and di-dehydrobacterioruberin) [13]. Furthermore, Raman spectroscopy has been employed to identify carotenoids in red halophilic archaea (*Halobacterium salinarum* NRC-1 and R1, and *Halorubrum sodomense*), confirming that the C50 carotenoid bacterioruberin is the predominant class of carotenoids among these archaea [53]. In this study, the major carotenoids in *H. paucihalophilus* TRM89021 were preliminarily identified as bacterioruberin and its derivatives, using UV and UPLC methods. Our findings are largely in agreement with those of previous studies, indicating a striking similarity in the carotenoids produced by various species of halophilic archaea, including *H. paucihalophilus* TRM89021. This provides another example case for the subsequent identification of carotenoids in halophilic archaea and contributes to further understanding of halophilic archaea.

### 4.3. Effects of Dietary Carotenoids Extracts on Egg Quality in the Hotan Black Chickens

Carotenoids are known to influence various aspects of egg quality in laying hens, including egg production, yolk color, eggshell thickness, and strength [23,25,54,55,56]. Our study investigated the effects of carotenoids produced by *H. paucihalophilus* TRM89021 on Hotan Black chickens and compared these effects with those reported in the literature. While carotenoid supplementation has been shown to improve egg production in some studies (*p* < 0.05) [24], others have found no significant effect [57]. Similarly, the impact on yolk color is inconsistent; astaxanthin and beta-carotene have been reported to enhance yolk color (*p* < 0.05) [24,57], yet not all studies support this finding [25,58,59,60]. In our study, the addition of *H. paucihalophilus* TRM89021-derived carotenoids led to a significant increase in the a* score from week 2 to week 5 in the BDG (*p* < 0.05). Contrary to some of the literature [25,58,59,60], our findings showed a significant increase in eggshell weight and size (*p* < 0.05), which could be attributed to improved absorption and conversion of calcium ions from the feed [61,62]. This enhancement in eggshell strength is useful for protecting eggs during transportation and crucial for increasing their resistance to damage [63], and increased egg weight is a desirable trait for consumers [64]. The significant increase in egg strength and weight at week 2 in the CDG compared with the BDG suggests that the carotenoids from *H. paucihalophilus* TRM89021 may have had a rapid and positive effect on these parameters. At week 5, the a* and b* scores were significantly lower in the CDG (*p* < 0.05). These differences highlight the dynamic effects of carotenoid supplementation over time and the potential for varying responses depending on the type of carotenoid and the duration of supplementation. The inconsistent effects of carotenoid supplementation on egg quality parameters underscore the need for further research to elucidate the mechanisms underlying these effects. Our study contributes to this body of knowledge by demonstrating the potential benefits of carotenoids produced by *H. paucihalophilus* TRM89021 on egg quality in Hotan Black chickens, particularly in terms of yolk color, eggshell strength, and weight.

Notably, carotenoids have strong antioxidant properties, including the C50 carotenoid bacterioruberin, a long-chain carotenoid with 13 conjugated double bonds and a terminal hydroxyl group, which is a potent free-radical scavenger [65]. Extracts of *Halorubrum*, *Haloarcula*, *Haloferax* [33], and *Haloarcula japonica* [66] bacterioruberin exhibited high antioxidant activity. Similarly, carotenoid extracts from *Hac. tebenquichense* SU10 [35] and *Halobacterium salinarum* [5] exhibited significant antioxidant potential. Assays for glutathione peroxidase, superoxide dismutase, total antioxidant capacity, and malondialdehyde levels are standard methods for assessing antioxidant potential [67]. Carotenoid supplementation in the diet of laying hens increased the activity of superoxide dismutase, catalase, and glutathione peroxidase in egg yolks and reduced malondialdehyde levels in laying hens. [23,24,57]. Therefore, the addition of carotenoids to feed improved the antioxidant capacity of egg yolks; however, the effect of C50 carotenoids as feed additives on egg yolk quality remains unclear. In this study, feeding carotenoids produced by *H. paucihalophilus* TRM89021 significantly increased the levels of GSH-Px and T-AOC in egg yolk. This indicates that the carotenoids produced by this strain demonstrated antioxidant activity similar to that of the carotenoids in the aforementioned study, implying that C50 carotenoids may have the same ability to improve the antioxidant ability of egg yolks when used as feed additives. Further research is warranted to elucidate the specific mechanisms by which these carotenoids exert their effects and to optimize their use in poultry feed.

### 4.4. Effects of Dietary Carotenoids on Plasma Biochemical Profiles in the Hotan Black Chickens

The addition of carotenoids to the diet affects the metabolic profile of laying hens. Plasma biochemical analyses have provided insight into the metabolic effects observed in laying hens. Fewer studies have reported the effects of carotenoid intake on plasma composition in laying hens: both lycopene and astaxanthin increased serum glucose levels in laying hens (*p* > 0.05), creatinine and cholesterol levels, and activities of ghrelin, glutamate, glutamate aminotransferase, and alkaline phosphatase varied with dose [68]. Currently, no standard exists for the normal range of plasma biochemicals in laying hens. Only one study referenced the blood biochemistry of broilers [69]. In that study, plasma TB and AST levels in the CDG were significantly higher (*p* < 0.05) and PHOS levels were significantly lower (*p* < 0.05). Plasma TB and AST levels together reflect whether the liver is damaged [69]. The values obtained in this study were within normal ranges, indicating that the liver was not damaged. Inorganic phosphorus may also be associated with kidney disease [70]. In that study report, there was no mention of the viscera, and determining the direct cause of the decrease in plasma inorganic phosphorus levels is currently impossible; further studies are still needed.

### 4.5. Effects of Dietary Carotenoids on Cecal Microbiota in the Hotan Black Chickens

The gut microbiota regulates nutrient absorption, curbs the proliferation of harmful bacteria, enhances growth and metabolism, and facilitates nutrient digestion and absorption. It also bolsters the defense of the animal against exogenous pathogens [71]. In chickens, the predominant intestinal phylum is *Firmicutes*, followed by *Bacteroidetes*, *Actinomycetes*, and *Pseudomonadota* [72]. Thick–walled phyla form the bulk of the microbial community in the gut environment of several birds [73]. A higher abundance of cecal unclassified *Bacteroidalesin* microorganisms was reported to be present in laying hens compared with broilers [74]. Unclassified *Bacteroidalesin* is are strongly associated with gut microbial–butyric acid/lipid metabolism and promote host gut health [75]. *Bacteroidota* are also important producers of B vitamins [76]. Previous studies have reported that astaxanthin can modulate the gut microbiota [77,78]. Dietary inclusion of lutein-rich prebiotics significantly elevated bifidobacteria and lactobacilli counts whereas it diminished populations of opportunistic pathogens such as *Anaplasma* spp. and *Clostridium* spp. [79].

In the present study, the dietary supplementation of Hotan Black chickens with carotenoids derived from *H. paucihalophilus* TRM89021 did not result in any significant changes in the cecal microbial composition at various taxonomic levels, including phylum, class, order, family, genus, and species (*p* > 0.05). Similarly, no significant alterations were observed in microbial operational taxonomic units (OTUs) in the cecum of laying hens, as assessed by alpha diversity indices such as ACE, Chao, and Shannon, as well as beta diversity metrics like Simpson, coverage, PCA, and PCoA (*p* > 0.05). This suggests that the addition of carotenoids to the diet did not significantly impact the cecal microbial diversity of Hotan Black chickens.

The dominant phyla identified in the cecum were *Bacteroidota* and *Firmicutes*, with the dominant genera and species being unclassified *Bacteroidales*. These findings are consistent with the previous literature on the dominant microbial phyla in the chicken cecum, indicating a stable and consistent microbial composition within the gut flora of these chickens. However, a non-significant increase in the abundance of *Bacteroidota* and *Firmicutes*, as well as of the dominant genera and species (unclassified *Bacteroidales*), was observed in the carotenoid-supplemented diet group (CDG) compared with the basal diet group (BDG) (*p* > 0.05), suggesting that carotenoids from *H. paucihalophilus* TRM89021 may have enhanced the population of beneficial bacteria in the cecum.

Interestingly, *Phocaeicola coprophilus* and *Paraprevotella clara* were exclusively detected in the CDG. These anaerobic, rod-shaped, Gram-negative bacteria are known to contribute to the production of short-chain fatty acids in the intestine [80], which can supply energy to the intestinal tract. Disruptions in Gram-negative bacteria have been reported to negatively impact the intestinal health of chickens. In particular, *Paraprevotella clara* is a potent trypsin-degrading commensal that aids in maintaining intestinal homeostasis and protects against pathogenic infections. Its colonization also inhibits lethal infections caused by mouse hepatitis virus [81]. These observations reflect the positive effect of dietary supplementation with carotenoids produced by *H. paucihalophilus* TRM89021 on cecal probiotics, potentially enhancing intestinal health and resistance to pathogens in Hotan Black chickens.

## 5. Conclusions

In this study, we isolated *H. paucihalophilus* TRM89021, a strain with a carotenoid yield of 20 mg/L, from a soil sample collected from the Pamir Plateau. Incorporation of *H. paucihalophilus* TRM89021-derived carotenoids into the diets of Hotan Black laying hens enhanced egg quality through increased laying capacity and elevated T-AOC levels in egg yolk, as well as improving cecal health by promoting a more abundant beneficial microbiota.

## Figures and Tables

**Table 1:** Nutritional value of the basal diet.

Guaranteed Value for Product Composition Analysis (%)		Raw Material
Crude protein ≥	16.50	Corn, soy meal, bran, corn protein powder, calcium phosphate, stone powder, sodium chloride, trace elements, v-itamins, amino acids, etc.
Crude fiber ≤	6.00	
Crude ash ≤	15.00	
Ca	3.00–4.40	
*p* ≥	0.50	
NaCl	0.15–0.80	
Methionine ≥	0.35	

**Table 2:** Effect of dietary carotenoid supplementation on production performance of Hotan Black chickens.

Items	BDG	CDG	*p* Value		
			BDG (Wk 2 vs. Wk 5)	CDG (Wk 2 vs. Wk 5)	BDG vs. CDG
Egg production (%) Wk 5	65.00 ± 8.29	67.50 ± 5.00	-	-	0.645
Eggshell color					
L* score					
Wk 2	74.95 ± 5.21	80.05 ± 1.37	0.461	0.066	0.177
Wk 5	71.26 ± 5.87	82.23 ± 0.63			0.082
a* score					
Wk 2	4.34 ± 0.60	4.30 ± 0.15	0.014	0.003	0.917
Wk 5	5.87 ± 0.16	3.13 ± 0.28			<0.001
b* score					
Wk 2	20.98 ± 1.67	17.55 ± 0.23	0.051	0.311	0.025
Wk 5	24.10 ± 0.10	16.12 ± 2.13			0.004
Egg strength (kg/m^2^)					
Wk 2	34.23 ± 1.10	44.27 ± 4.72	0.012	0.830	0.023
Wk 5	38.83 ± 1.47	43.32 ± 5.40			0.238
Eggshell thickness (mm)					
Wk 2	0.29 ± 0.02	0.33 ± 0.05	0.161	0.933	0.217
Wk 5	0.32 ± 0.03	0.33 ± 0.05			0.919
Egg-shaped index					
Wk 2	0.78 ± 0.02	0.74 ± 0.01	0.657	0.494	0.058
Wk 5	0.77 ± 0.05	0.76 ± 0.03			0.783
Egg weight (g/egg)					
Wk 2	46.38 ± 2.97	59.16 ± 4.57	0.041	0.665	0.012
Wk 5	52.96 ± 4.45	56.79 ± 7.51			0.465
Yolk color					
Wk 2	8.33 ± 1.15	9.67 ± 0.58	0.148	0.101	0.148
Wk 5	9.67 ± 0.58	10.67 ± 0.56			0.101
Haugh unit					
Wk 2	81.75 ± 9.87	62.03 ± 5.99	0.277	0.166	0.042
Wk 5	74.24 ± 3.08	70.18 ± 5.82			0.346
Feed consumption (g)					
Wk 2	186 ± 5.29	194.33 ± 5.13	0.648	0.222	0.122
Wk 5	187.67 ± 2.52	186.67 ± 7.64			1.000
Feed conversion ratio (g/g)					
Wk 2	4.02 ± 0.30	3.29 ± 0.20	0.091	0.905	0.024
Wk 5	3.55 ± 0.22	3.34 ± 0.60			0.601

Note: Data are expressed as mean ± standard error (SE) for each group, and the *p* values represent the results of statistical comparisons between the BDG and CDG. BDG (Wk 2 vs. Wk 5): eggs produced by laying hens in the BDG group, with a significant difference between eggs at weeks 2 and 5. CDG (Wk 2 vs. Wk 5): eggs produced by laying hens in the CDG group, with a significant difference between eggs at weeks 2 and 5. BDG vs. CDG indicates the significance of comparing eggs produced by laying hens in both groups simultaneously (Wk 2 or Wk 5). During the experimental period, no animals experienced accidental deaths.

**Table 3:** Effect of dietary carotenoid supplementation on the antioxidant capacity of egg yolks.

Items	BDG	CDG	*p* Value		
			BDG (Wk 2 vs. Wk 5)	CDG (Wk 2 vs. Wk 5)	BDG vs. CDG
CAT (µmol/min/g)					
Wk 2	83.027 ± 10.582	88.022 ± 11.657	0.048	0.004	0.612 0.153
Wk 5	55.749 ± 13.058	40.644 ± 7.066			
GSH-Px (nmol/min/g)					
Wk 2	92.026 ± 7.433	88.121 ± 4.497	0.074	<0.001	0.480 0.050
Wk 5	127.988 ± 24.871	170.432 ± 8.843			
MDA (nmol/g)					
Wk 2	8.000 ± 0.733	8.240 ± 0.591	0.560	0.137	0.686 0.279
Wk 5	8.417 ± 0.856	9.209 ± 0.684			
SOD (U/g)					
Wk 2	51.832 ± 6.193	51.068 ± 1.935	0.543	0.393	0.848 0.667
Wk 5	54.736 ± 4.354	53.251 ± 3.444			
T-AOC (µmol Trolox/g)					
Wk 2	0.073 ± 0.017	0.060 ± 0.019	0.038	<0.001	0.416 0.013
Wk 5	0.166 ± 0.050	0.345 ± 0.052			

Note: Data are expressed as mean ± standard error (SE), and *p* values represent statistical comparisons of the CDG group at weeks 2 and 5. Abbreviations: total antioxidant capacity (T-AOC), superoxide dismutase (SOD), malondialdehyde (MDA), glutathione peroxidase (GSH-Px), and catalase (CAT).

**Table 4:** Effect of dietary carotenoid supplementation on serum biochemical indices in laying hens.

Items	BDG	CDG	*p* Value
Albumin–globulin ratio (A/G)	0.687 ± 0.038	0.583 ± 0.04	1.000
Globulin (GLOB) (g/L)	30.300 ± 4.723	40.233 ± 2.200	0.311
Amylase (AMY) (U/L)	328.667 ± 39.716	294.667 ± 42.736	0.593
Total bilirubin (TB) (umol/L)	4.867 ± 0.577	11.500 ± 1.411	0.035
Glucose (GLU) (mmol/L)	9.190 ± 1.249	10.933 ± 0.793	0.482
Albumin (ALB) (g/L)	20.767 ± 1.400	23.400 ± 1.464	0.967
Total protein (TP) (g/L)	51.033 ± 5.950	63.667 ± 3.288	0.517
Urea nitrogen (BUN) (mmol/L)	1.163 ± 0.035	1.167 ± 0.069	0.775
Creatine kinase (CK) (U/L)	1348.667 ± 206.986	1428.000 ± 143.123	0.950
Inorganic phosphorus (mmol/L)	3.155 ± 1.824	2.673 ± 0.032	0.004
Aspartate aminotransferase (AST) (U/L)	175.333 ± 4.509	189.667 ± 26.764	0.028

Note: Data are expressed as mean ± standard error (SE), and *p* values represent statistical comparison between the BDG and CDG groups.

**Table 5:** Cecal microbial abundance at the OTU level in laying hens.

Estimators	BDG	CDG	*p* Value
ACE	478.37 ± 21.47	447.27 ± 28.03	0.4469
Chao	485.63 ± 18.98	455.06 ± 26.58	0.4131
Coverage	0.99699 ± 0.00	0.99652 ± 0.00	0.1806
Shannon	4.607 ± 0.15	4.3148 ± 0.13	0.2271
Simpson	0.032655 ± 0.01	0.037817 ± 0.01	0.6769
Sobs	448.67 ± 20.90	406.67 ± 29.10	0.3172

Note: This table presents the estimated microbial abundance within the cecal microbiome of laying hens in the BDG and CDG groups. The estimators used included ACE (abundant coverage estimator), Chao (Chao richness estimator), coverage (proportion of species observed), Shannon (Shannon diversity index), Simpson (Simpson’s diversity index), and Sobs (species observed). *p* > 0.05 indicates that the difference between groups was not significant.

## Data Availability

Raw reads of bacterial 16S rDNA gene sequencing are available in the NCBI Sequence Read Archive database (Accession Number: PRJNA1168399). Other data that support the findings of this study have not been deposited in an official repository; however, they are available from the authors upon request.

## Supplementary Materials

The following supporting information can be downloaded at https://www.mdpi.com/article/10.3390/ani14233470/s1. Figure S1: Effect of dietary carotenoid supplementation on the bacterial composition of the cecum; Figure S2: Comparison of species levels; Figure S3: Phylum, Class, Order, Family, Genus, and Species Composition; Table S1: 16S rRNA gene amplification conditions.
