# Supplementary material for: Carotenoids from Halophilic Archaea: A Novel Approach to Improve Egg Quality and Cecal Microbiota in Laying Hens
Source: Animals (Basel). 2024 Dec 1;14(23):3470. doi: 10.3390/ani14233470 (PMC11640664; doi:10.3390/ani14233470)
Supplement: Supplementary file 1 [file animals-14-03470-s001.zip › Supplementary table.pdf]

Table S1 16S rRNA gene amplification conditions

Initial denaturation	Denaturation	Annealing	Extension	Extension
95℃	95℃	55℃	68℃	72℃
5 min	40 s	2 min	2 min	8 min
1 cycle		30 cycles		1 cycle
