# Supplementary figures and images for: Carotenoids from Halophilic Archaea: A Novel Approach to Improve Egg Quality and Cecal Microbiota in Laying Hens
Source: Animals (Basel). 2024 Dec 1;14(23):3470. doi: 10.3390/ani14233470 (PMC11640664; doi:10.3390/ani14233470)

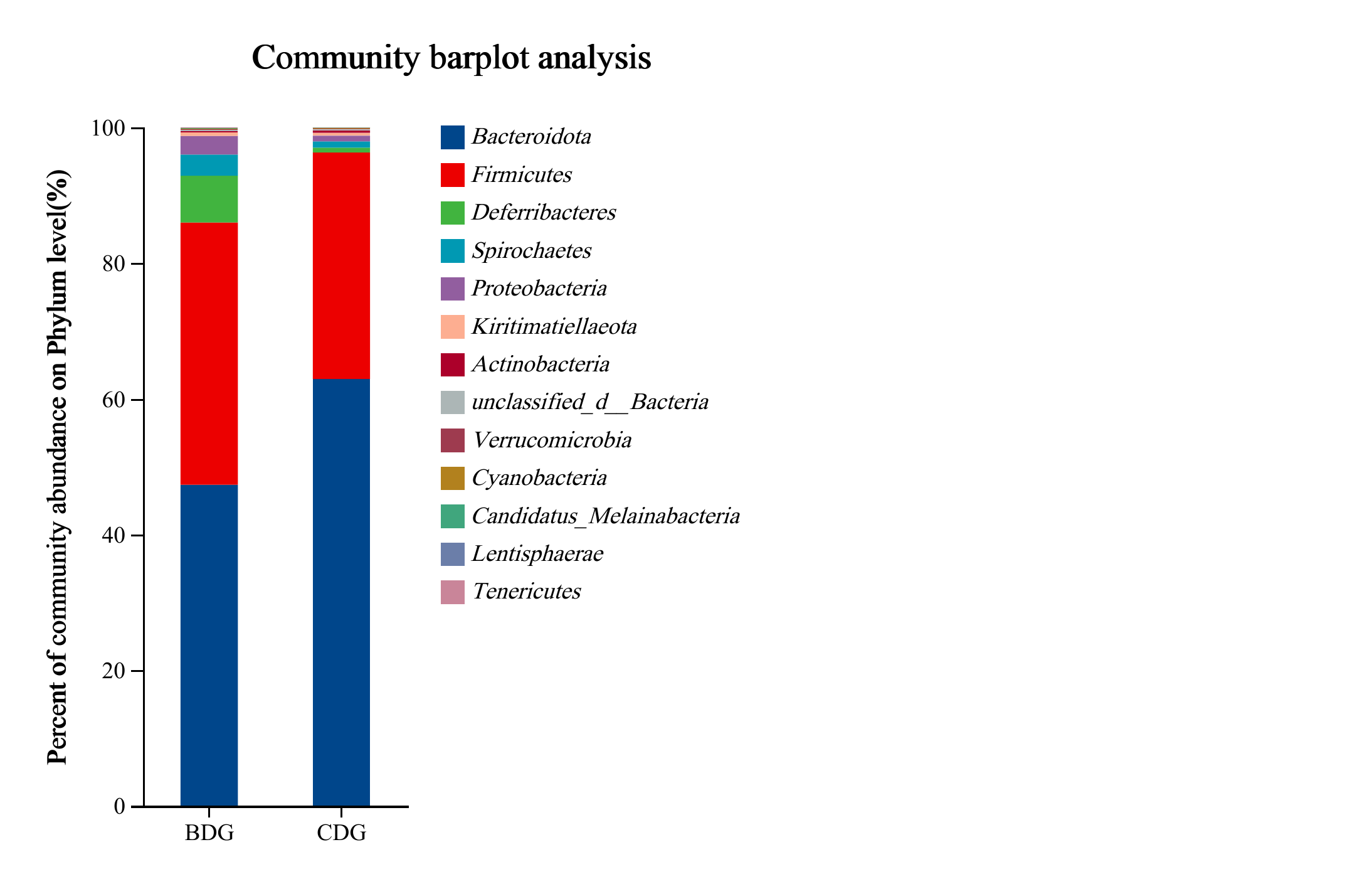

Supplement: Supplementary file 1 [file animals-14-03470-s001.zip › Figure S1 (a).tiff]

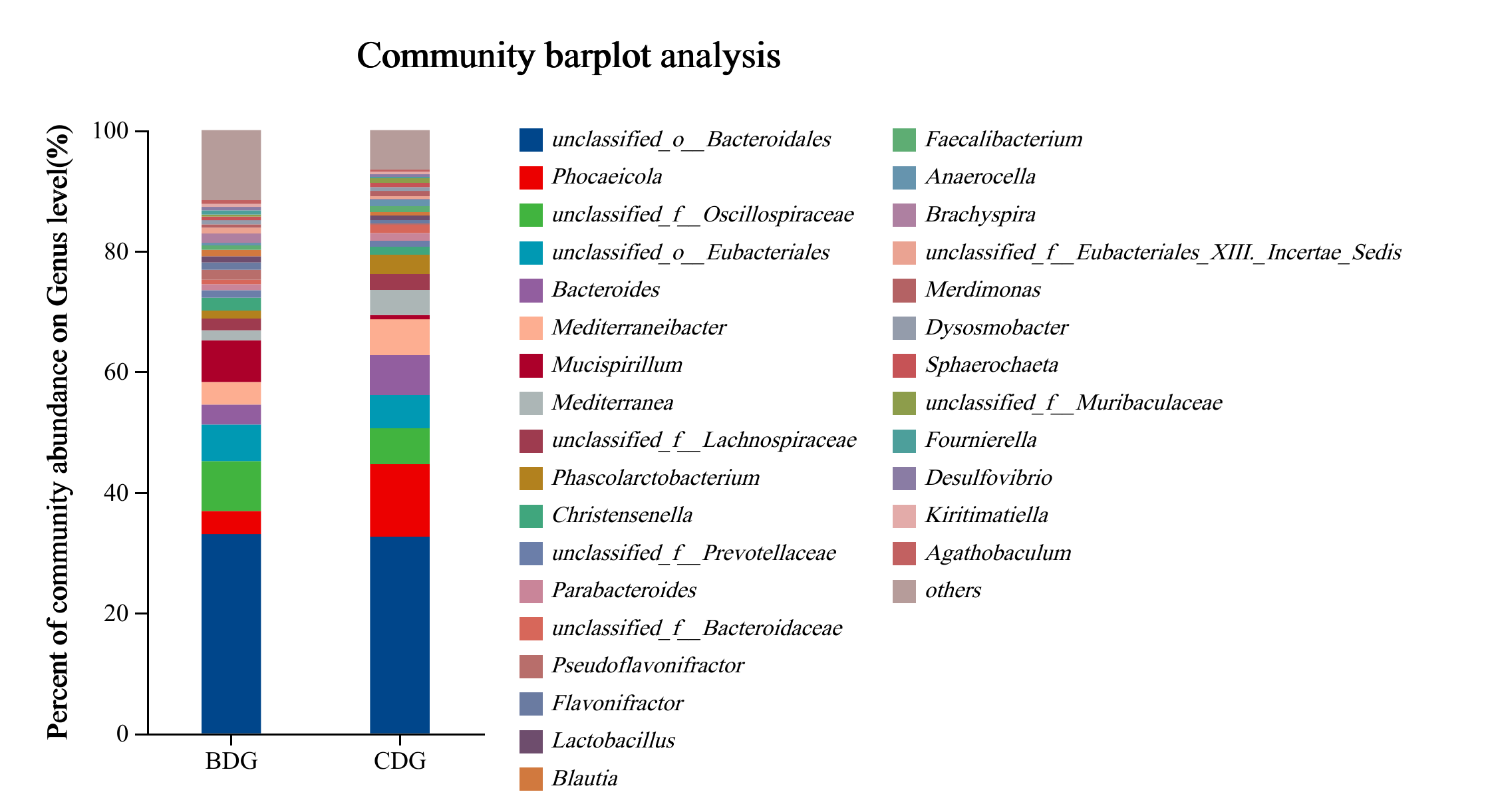

Supplement: Supplementary file 1 [file animals-14-03470-s001.zip › Figure S1 (b).tiff]

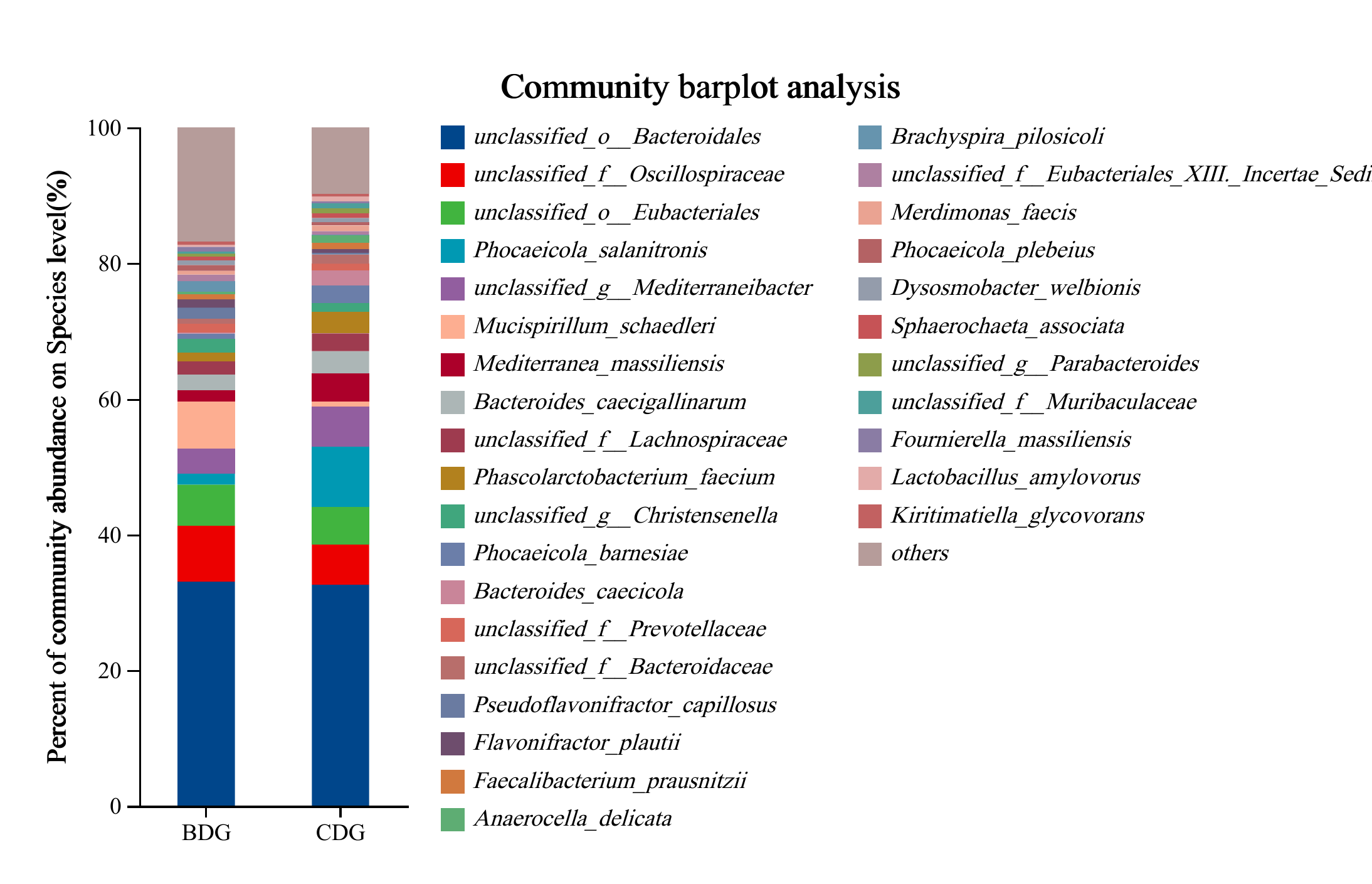

Supplement: Supplementary file 1 [file animals-14-03470-s001.zip › Figure S1 (c).tiff]

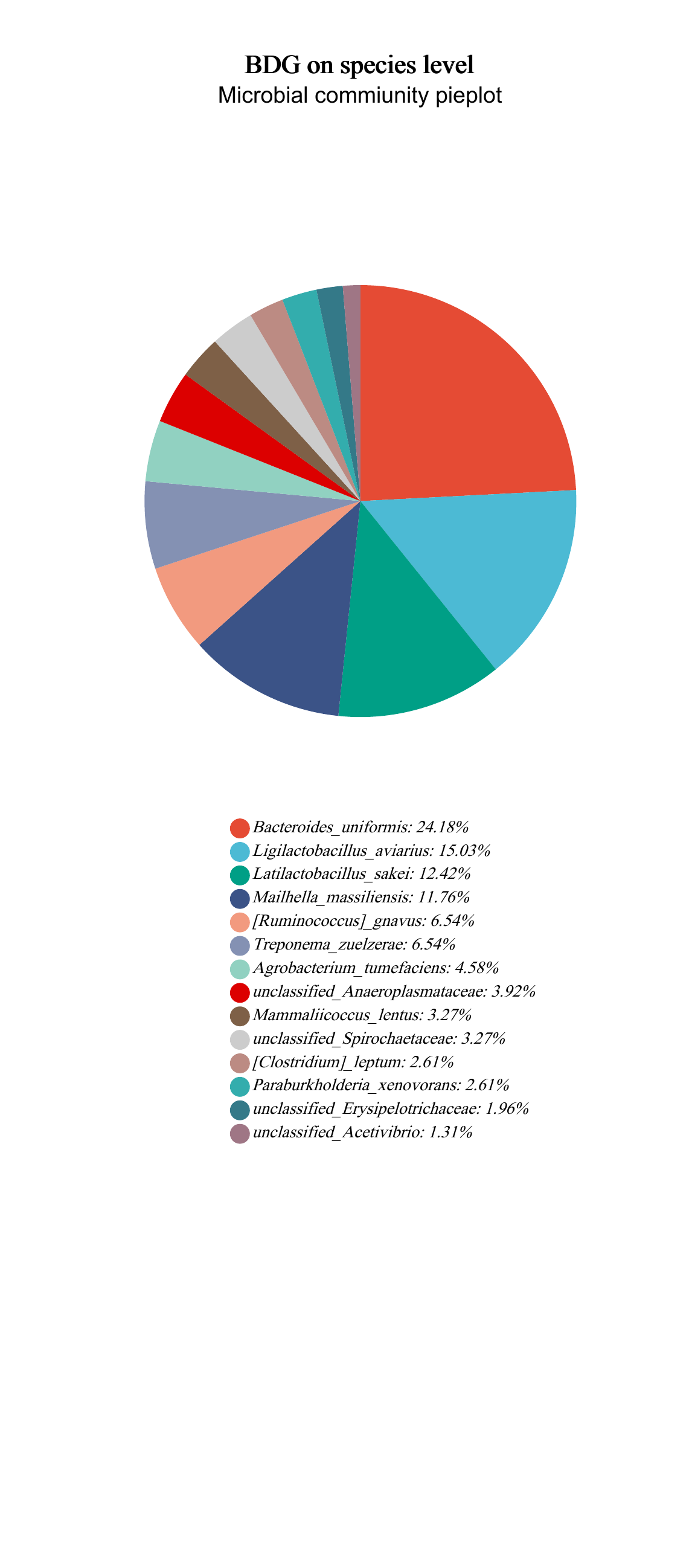

Supplement: Supplementary file 1 [file animals-14-03470-s001.zip › Figure S2 (a).tiff]

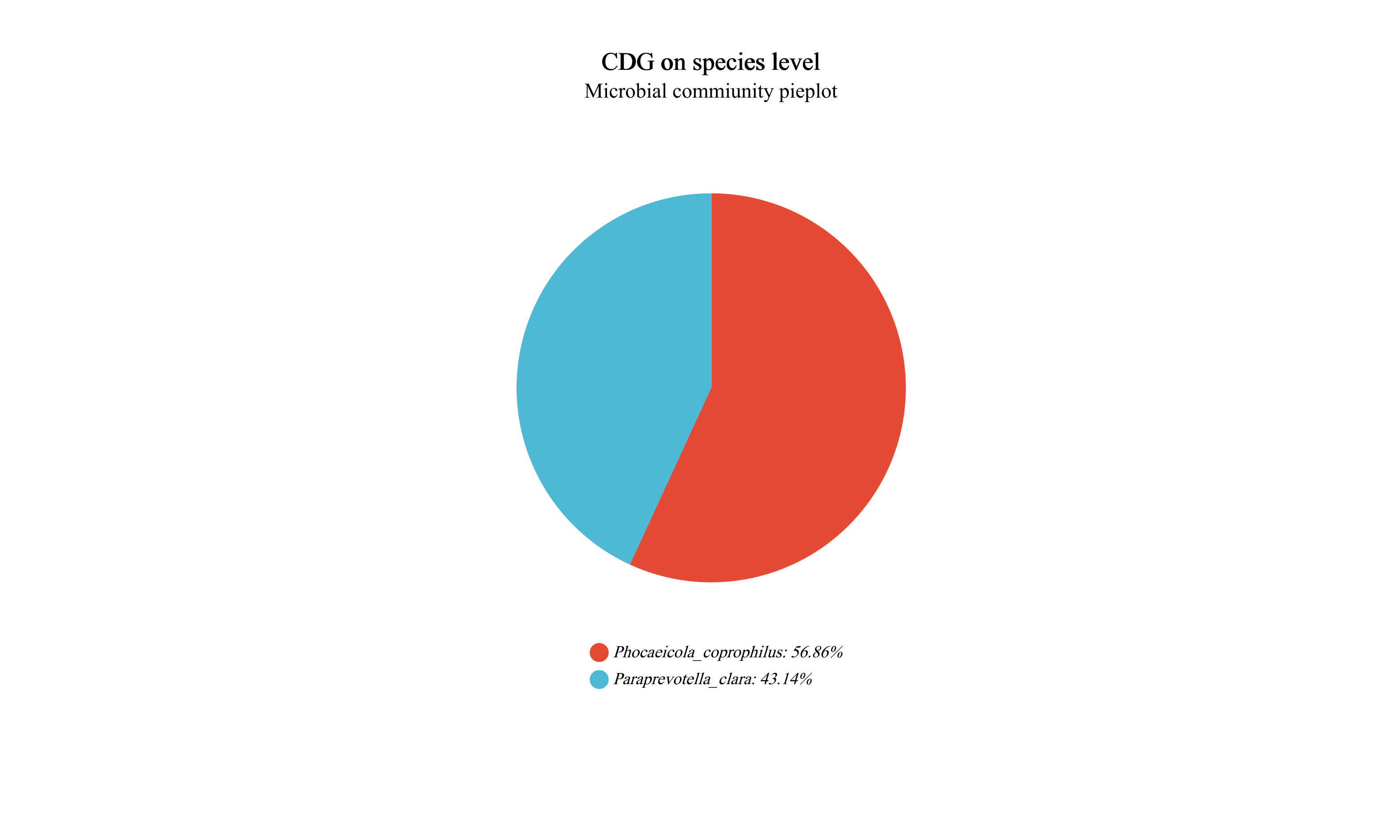

Supplement: Supplementary file 1 [file animals-14-03470-s001.zip › Figure S2 (b).tiff]

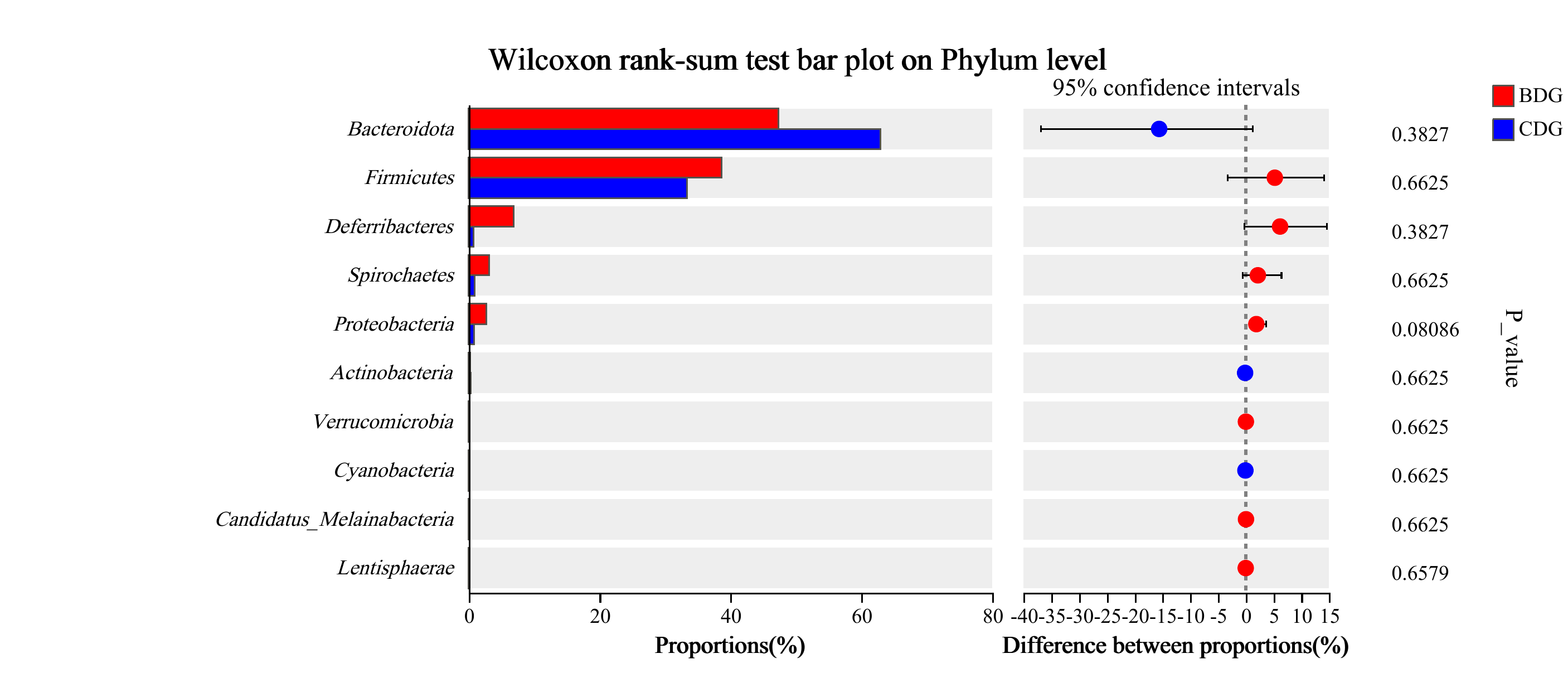

Supplement: Supplementary file 1 [file animals-14-03470-s001.zip › Figure S3 (a).tiff]

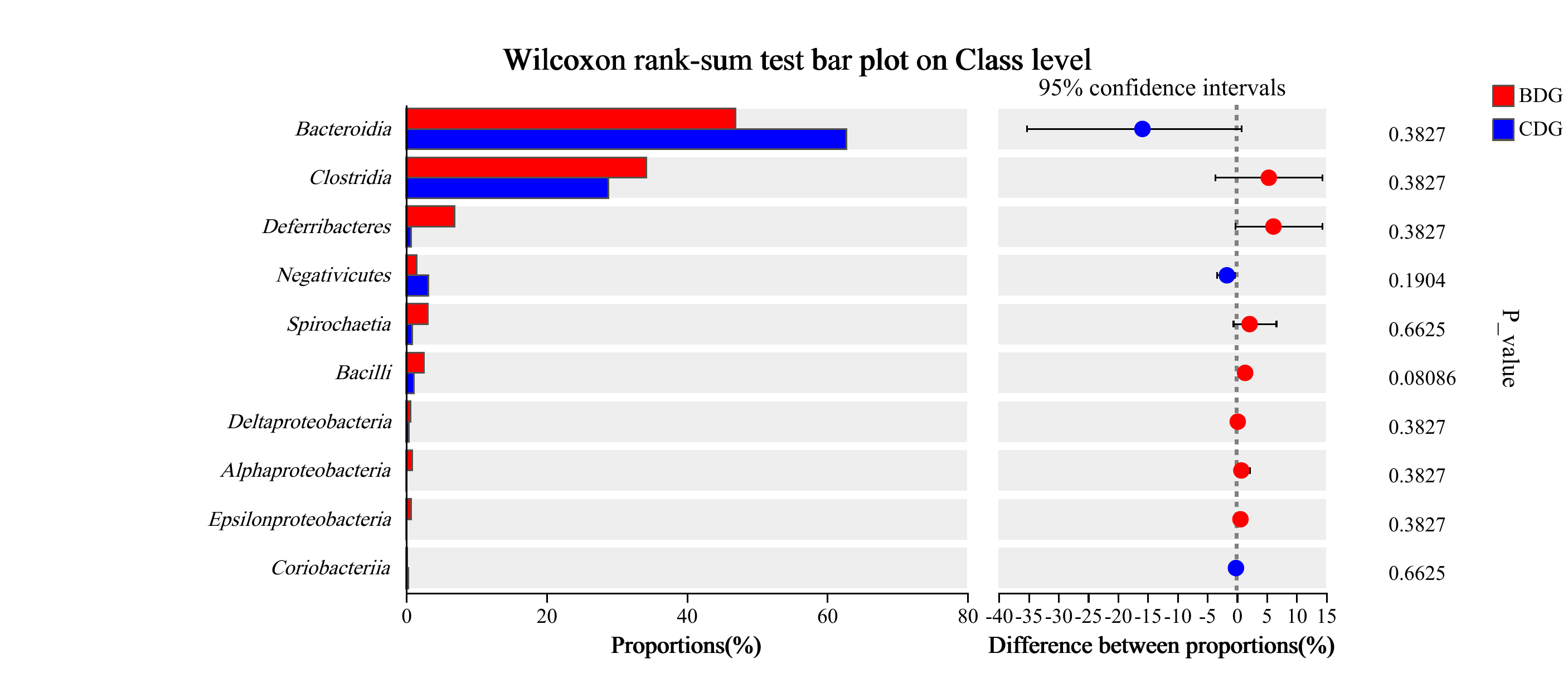

Supplement: Supplementary file 1 [file animals-14-03470-s001.zip › Figure S3 (b) .tiff]

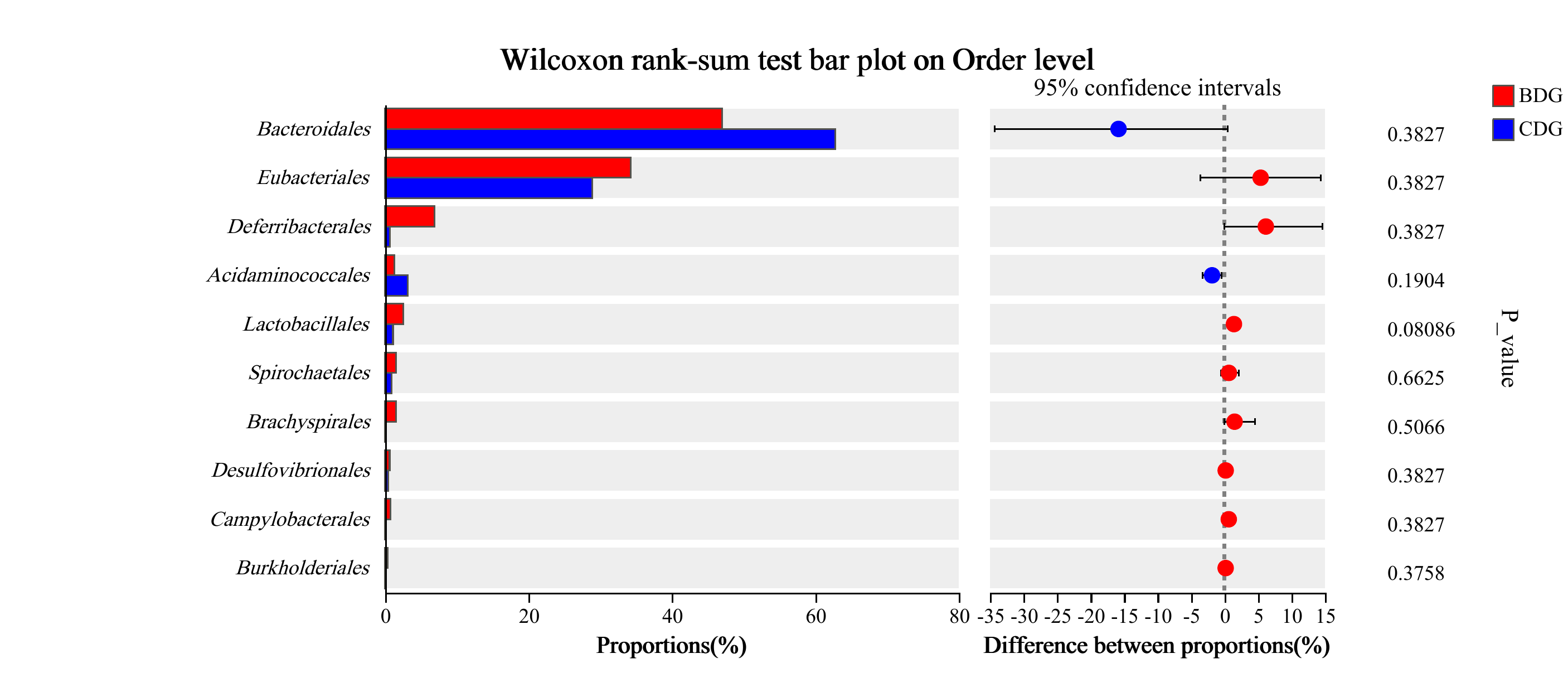

Supplement: Supplementary file 1 [file animals-14-03470-s001.zip › Figure S3 (c).tiff]

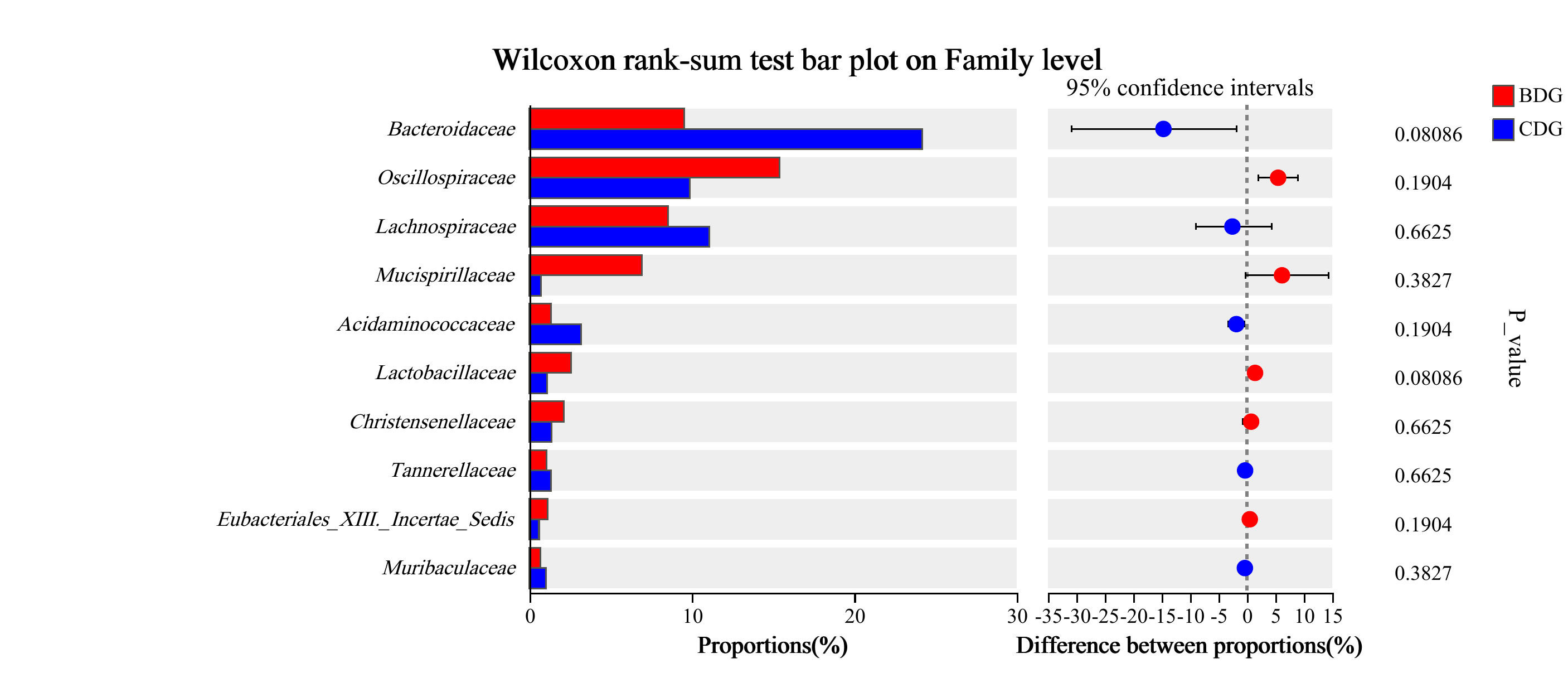

Supplement: Supplementary file 1 [file animals-14-03470-s001.zip › Figure S3 (d).tiff]

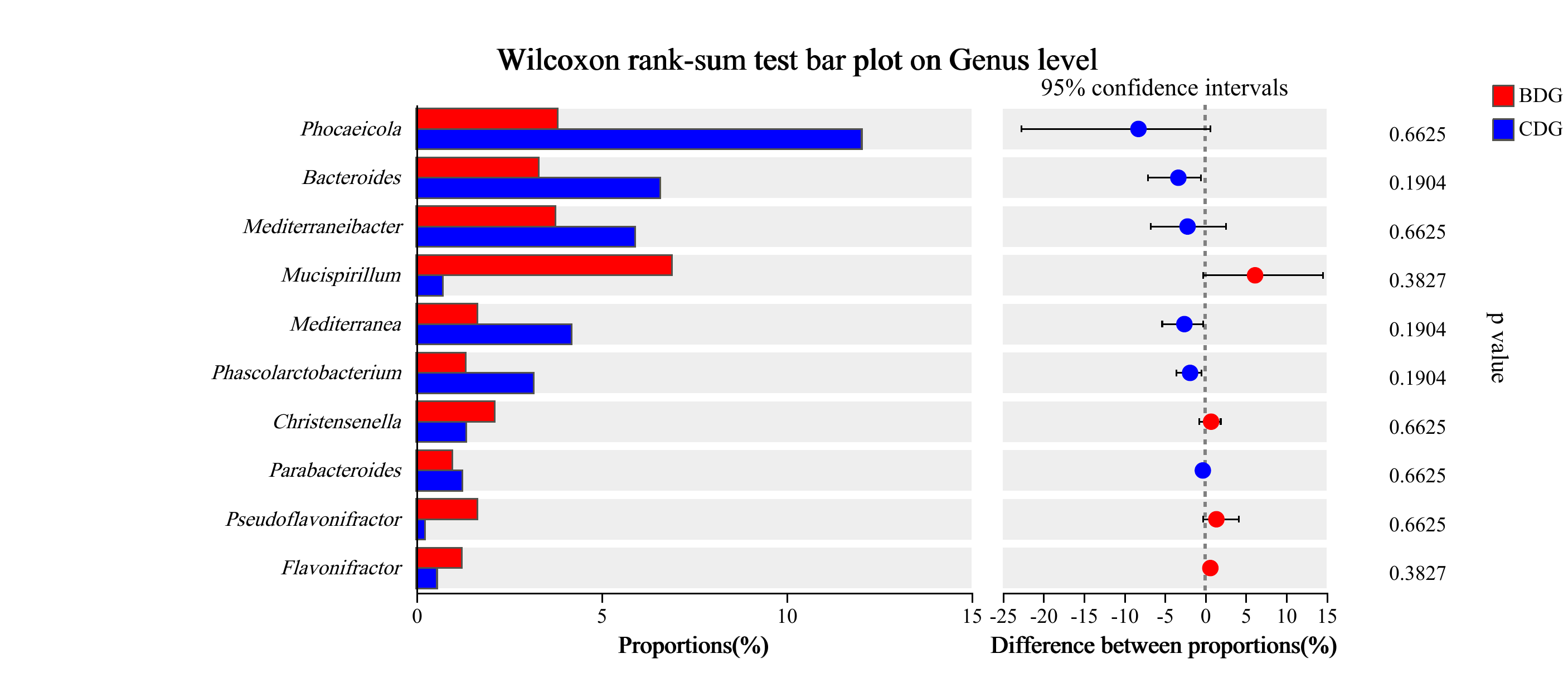

Supplement: Supplementary file 1 [file animals-14-03470-s001.zip › Figure S3 (e).tiff]

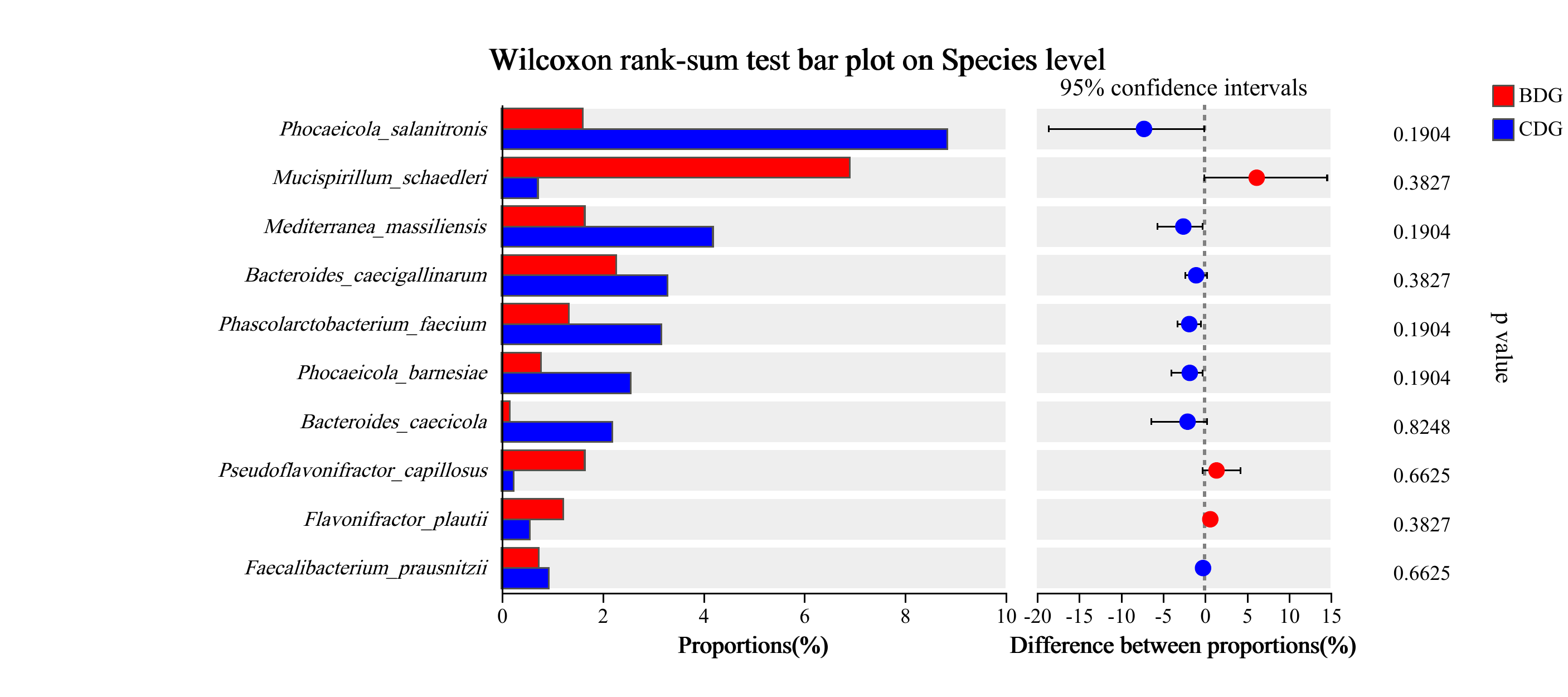

Supplement: Supplementary file 1 [file animals-14-03470-s001.zip › Figure S3 (f).tiff]
